# Evaluation of the iron, zinc, and folic acid stability in fortified wheat flour storage and its impact on quality indicators

**DOI:** 10.1186/s40795-025-01213-z

**Published:** 2025-12-18

**Authors:** Abebe Bitew Zegeye, Abebe Ayelign, Alebachew Habte Gezmu, Chere Tadesse, Kelemua Tesfaye, Misganaw Andualem, Zeweter Abebe Sime

**Affiliations:** 1 Center for Food Science and Nutrition, Addis Ababa University, P. O. Box; 1176, Addis Ababa, Ethiopia; 2 Nutrition, Environmental health and Non Communicable Diseases Research Directorate, Ethiopian Public Health Institute, P.O. Box,1242, Addis Ababa, Ethiopia; 3 Ethipina Drug and Food Administration, P.O. Box, 5681, Addis Ababa, Ethiopia

**Keywords:** Wheat flour, Fortification, Iron, Zinc, Folic acid, Stability study

## Abstract

The fortification of wheat flour with micronutrients is regarded as an effective strategy for preventing micronutrient deficits in the general population. Therefore, this study was intended to evaluate the storage stability of iron, zinc, and folic acid in fortified wheat flour, as well as the overall quality properties of flour during storage. Wheat flour was fortified (with zinc oxide 80 mg/kg, iron sodium EDTA 30 mg/kg, and 2 mg/kg folic acid) and stored over a 6-month period in a polyethylene bag. Iron was analyzed based on the AOAC official method 944.02, dry ash technique, ultraviolet-visible reading spectrophotometer. Zinc was determined using micro plasma atomic emission spectroscopy and the folic acid content was determined using HPLC.

Micronutrients remain stable during storage. Zinc and iron showed no significant losses at stored temperatures. The percentages of zinc and iron retention ranged from 96.1 to 100%; however, folic acid showed significant losses, and the percentages of retention ranged from 68.5 to 87.8%. Peroxide value was increased from 0 to 0.1484milliequivalents per kilogram (mEq/kg) of fat during storage, and pH decreased from 6.23 to 5.38 during the period of six months of storage. The moisture content did not exhibit any significant changes. In conclusion, fortification of iron, zinc, and folic acid in wheat flour remains stable during storage for six months. Fortification is a good technique to lower population micronutrient deficits.

**Supplementary Information:**

The online version contains supplementary material available at 10.1186/s40795-025-01213-z.

## Introduction

Micronutrients (vitamins and minerals) are essential for proper cellular and molecular function. Furthermore, micronutrients are an essential part of the diet and are necessary in trace amounts; a shortage of them can have severe effects on health [1]. Micronutrient deficiencies are a serious public health issue around the world, particularly in low- and middle-income countries (LMICs), owing to insufficient food consumption, a lack of dietary diversity, and poor nutrient absorption due to infection, inflammation, and chronic illness, and especially prevalent in infants and pregnant women [1].

WHO report in 2015, showed that approximately 2 billion individuals (more than 30% of the world’s population) are anemic, with many suffering from iron deficiency [2].

Zinc deficiency was also related to 116,000 deaths in children under the age of five [3]. Folic acid deficiency is known to cause neural tube defects, megaloblastic anemia, and cancer [4], and many of these birth problems could be addressed by taking folic(Blencowe et al., 2018.

An estimated 260,100 deliveries were influenced by neural tube defects (NTDs) in 2015, with over 75% of neural tube defect (NTD)-affected infants leading to under-five mortality [5].

According to CDC Nutrition [6], all women capable of becoming pregnant should get 400 micrograms of folic acid every day. According to the National Institute of Health [7], the(daily recommended intake) DRI of zinc for 6-month- to 3-year-olds (3 mg), pregnant women (11 mg), breastfeeding women (12 mg), and breastfeeding teens (13 mg) is required. The DRI of iron for infants 7–12 months (11 mg), children 1–3 years (7 mg), pregnant teens and pregnant women (27 mg), breastfeeding teens (10 mg), and breastfeeding women (9 mg) is required [8].

According to the 2013 Ethiopian Nation Food Consumption Survey report [9], Ethiopian men, women, and children consumed very little enriched cereal, whole grain cereal, legumes, and nuts.

Micronutrient deficiency is one of the major public health problems in Ethiopia, with women and children being especially at risk [10]. According to the Ethiopian Public Health Institute Micronutrient Survey report (2016), the prevalence of anemia adjusted for altitude among pre-school children, school age children and non-pregnant women of reproductive age was 34.4%, 25.6% and 17.7%, respectively. The national prevalence of zinc deficiency was 35% in pre-school age children and higher (40.3%) in children 12 to 23 months, 36% in school age children and 34% in women of reproductive age. Among women reproductive age, vitamin B12 deficiency is 15.1%, serum folate deficiency is 17.3%, and RBC folate or red blood cell folate deficiency is 32% [10].

Another study also exhibited, In Ethiopia, 31.7% of pregnant women were anemic [11], and additional research in Ethiopia confirmed a significant prevalence of anemia during pregnancy [12]. According to the 2016 Ethiopian Demographic Health Survey, 37.51% of reproductive-age women were anemic [13]. In poor and middle-income countries, anemia results in 18% of perinatal mortality, 19% of preterm births, and 12% of low birth weight [13]. Zinc deficiency was found in 9.5% of developed countries and 33.5% of developing.in Ethiopia reveals that zinc deficiency was present in 59.9% and 38.4% of pregnant women and children, respectively [14].

Three major strategies are used to combat micronutrient deficiencies: food fortification, food diversification, and supplementation [15]. Food fortification is widely applied to alleviate micronutrient deficiency [16]. By adding micronutrients to frequently consumed food products, it allows for reaching target populations whose daily dietary needs for micronutrients are not routinely met while minimizing the risk of overconsumption among groups whose status is normal. The 76th World Health Assembly had issued a resolution to support fortification efforts [16, 17]. Lare Food fortification displays the advantages of being well accepted by populations, with interesting cost-efficacy ratios. As Global Alliance for Improved Nutrition report, for every US$1 invested in fortification, it generates US$27 in economic return [18].

Being a popular and common staple food in many countries, wheat flour is often considered one of the most suitable vehicles for multi-micronutrient fortification [19–22]. Food fortification in staple foods, on the other hand, is widely regarded as the most cost-effective intervention for addressing micronutrient deficiencies, particularly in developing countries including Ethiopia [19, 21, 22]. Two national surveys, “The Ethiopian National Food Consumption Survey [9] and “The Ethiopian National Micronutrient Survey” [10], were carried out. These investigations revealed the existence of a significant burden of micronutrient deficiencies. Recognizing this, the Ethiopian government has committed to fortifying salt with iodine, vegetable oil, and wheat flour with micronutrients [23].

Also, the Ethiopian standard council approved the mandatory fortification of wheat flour with zinc, folic acid, and vitamins B1, B2, B3, B6, and B9 in June 2022. Furthermore, the study’s design was grounded in scientific data to ensure an evidence-based approach. Wheat flour and vegetable oils are the staple foods selected in Ethiopia for mandatory fortification. This selection was based on their wide consumption among all population groups. Based on the 2021–2024 National Food and Nutrition Strategy Baseline Survey, the coverage of fortified salt, wheat flour and edible oil is (98%), (29%) and (89%) respectively in Ethiopia at the national [24].

The success of a fortification program is dependent on the stability of micronutrients and the food to which they are added. The fortification’s stability is influenced by physical and chemical factors such as heat, moisture, air, or light, and acid or alkaline environments encountered during food processing, packaging, distribution, or storage [25]. According to the study [26], the degree of fortification, storage, extraction level, baking, as well as association or lack thereof with other chemical components, appear to affect the fortification out comes [26]. Due to the fact that fortified flours might experience physicochemical changes while being stored and processed.

Physicochemical changes can also occur in fortified flours during storage due to iron’s pro-oxidant, the metals’ reactivity, and their sensitivity to the oxidizing and reducing effects of folic acid [27]. Moreover, there is limited is study on the stability of zinc, iron, and folic acid micronutrients in fortified wheat flour in country, Ethiopia. Hence, the objective of this study was to investigate iron, zinc, and folic acid retention stability in fortified wheat flour over a six-month storage periodand to evaluate its impact on key quality indictors including peroxide value, pH, and moisture content.

## Materials and methods

### Study setting

The research was carried out at the Center for Food Science and Nutrition Laboratory, Addis Ababa University (AAU), the Ethiopian Food and Drug Administration (EFDA) laboratory, and at food science and nutrition laboratory of Ethiopian Public Health Institute (EPHI).

### Study design

Study was carried out between June 2022 and June 2023 time period and A complete randomized controlled design was used to evaluate storage and its impact on quality indicators using the prepared standard fortification nutrient level.

### Fortification protocol of wheat flour with NaFeEDTA, ZnO and folic acid

The wheat flour sample used in this study was collected from the KOJJ food processing company and Extraction rate was 79% by mass (information was obtained from processor. The sample of wheat flour had a moisture content of 12.23 ± 0.11%, a fat content of 1.26 ± 0.09%, and an ash content of 0.62 ± 0.02%.

Zinc, iron, and folic acid fortified wheat flour was developed based on Ethiopian standard [28], as shown in Table 1. Zinc oxide and NaFeEDTA USP pharmaceutical grade was obtained chemical service (UK) LTD and folic acid food grade made Switzerland was used in this study.

**Table 1:** Requirements for levels of micronutrients used for the fortification of wheat flour

Fortificant compound	Level of fortification(mg/kg)
Zinc oxide, 99%	80 mg/kg
NaFeEDTA, 13% Fe	30 mg/kg
Folic acid, 90%	2 mg/kg

Fortification was performed in compliance with the Ethiopian Standard Agency’s recommendation of ES 6132:2021: fortified wheat flour specification [28]. Accordingly, 8.4 mg of folic acid (considering 10% loss during fortification by light), 700.53 g of NaFeEDTA, and 304.81 g of zinc oxide were weighted to achieve 2 ppm of folic acid, 30 ppm of iron, and 80 ppm of zinc. The nutrients were thoroughly mixed using spatula until homogenized. The mixed nutrients were fractionated into 6 parts, then each fraction (168.96 mg) of the fortiificant was added to one fractionated (500 gram) portion of wheat flour and a total of three kilograms of wheat flour were used.

After adding the fortifiers in to the flour, the mixture was homogenized and blended for ten minutes using a blender (Black and Decker made in made in England and model number is SC300). Following that, all fractionated flour containing added nutrients was mixed and homogenized for 10 min before sieving using 2-micrometer sieves and this phase was performed five times, The fortified flours were then packed in polyethylene bag containers to conduct stability study it was used to determine folic acid, zinc, and iron contents of the fortified flour that was considered as a base -line or zero month.

### Storage stability of wheat flour fortified with iron, zinc and folic acid

The fortified flour was divided into approximately 500 g and was packed in a polyethylene bag and stored in incubators set at 25 °C, 35 °C, and 45 °C. For each settled temperature and we stored approximately 500 g of fortified flour in two polyethylene bags, and we utilized one packed fortified flour for a 3-month period and the second one for a 6-month period. The storage temperatures were set to represent the different temperature ranges existing in different parts of Ethiopia. Extreme conditions (45 °C), in regions such as Afar and Somalia, moderate conditions (35 °C) in regions such as SNNPR and Oromia, and mild conditions (25 °C) observed in the highlands.

 The level of iron, zinc and folic acid was determined at 0, 3, and 6 months of storage and storge period was based on the following protocol [29–31].

At each month, one new packed flour was taken from each treatment and discarded after analyses. The percentage of micronutrients retained in fortified wheat flour at sixth-months of storage was calculated according to the procedure reported by [32].

$$\%\;R\;=\;100\;-\;\frac{concentration\;of\;0\;month-concentration\;of\;6\;th\;month}{concentration\;of\;0\;month}\;\ast\;100$$

### Iron analysis in food

According to the 20th edition of the AOAC, 2016 (AOAC official method 944.02, dry ash technique, ultraviolet-visible recording spectrophotometer model UV-1800, Shimadizu used to determined iron. Five grams of food were placed in a porcelain dish and ignited at 550 degrees Celsius in furnace, for three hours. After cooling, the dish was filled with 5 ml of hydrochloride, and the upper part of the dish was allowed to be washed with acid before being dried on a steam bath. Following that, the residue was dissolved in 2 ml of HCl acid and heated for 5 min in a steam bath, and the undissolved residue was filtered using ashless paper then diluted with 100 ml of water. After 5 min, 10 ml of the aliquot was pipetted into a 25 ml volumetric flask, followed by 1 ml of hydroxylamine hydrochloride (37% made in India) solution (10 g of hydroxylamine hydrochloride (Sigma-Aldrich) dissolved in 100 ml of water) and 5 ml of acetate buffer solution (8.3 gram of anhydrous sodium acetate that dried at 100 °C and 12 ml of acetic acid diluted in 100 ml by water) after 5 min. 1 ml of o- (0.1 g of o-rthophenanthroline dissolved in 80 ml of water that boiled at 80 °C, cooled, and diluted to 100 ml) was added, and the volume was diluted before reading at 510 nm and converting the iron concentration using the calibration curve.

### Standard preparation

3.512 g Fe (NH_4_)_2_(SO_4_)_2_.6H_2_O was weighted and dissolved in 500 ml of a volumetric flask with two drops of HCl as a stock solution. The 100-mg/kg intermediate solution was generated by adding 10 ml to 100 ml. Finally, each calibration point was prepared by adding 2 ml of HCl, and 6 calibration points such as 0, 0.5, 1, 2, 3, 4, and 5 mg/kg were prepared by taking 0, 0.5, 1, 2, 3, 4, and 5 ml, respectively, from 100 mg/kg.

### Zinc determination from food

Zinc concentration was determined following method developed by Boen & Pallone [33]. 0.60 g of flour was weighted and digested with a mixture of HNO3(70% made India) and H_2_O_2_ (30% m/v made India) in a digestion block. Concentrated HNO_3_ (4 ml) and of H_2_O_2_ (2 ml) (30% m/v) of were used for mineralization, and 4 ml of HNO_3_ was added in the tubes. A small funnel was placed on the tube to allow the HNO_3_ to reflux and placed in the digestion block (Foss tectar ^tm^), heated to 100 °C for 60 min, and cooled, and the H_2_O_2_ was carefully added. Then the block temperature was raised to 130 °C. The entire mineralization process took 4 h to finish. The mineralized samples were then quantitatively transferred to volumetric flasks (25 ml), and the capacity of each was filled with a 0.01 mol/L solution of HNO_3_. Standard solution having points 0, 0.5, 1, 2, 3, 4, 6, and 8 mg/kg was used for calibration and 0.01 mol/L HNO_3_ solution was used to make up the standard. Micro plasma atomic emission spectroscopy (MP-AES), Agilent model,4200/4210 was used to analyze the standard solutions and samples at 213.857 nm.

### Folic acid analysis in food

The folic acid content was determined using HPLC [34],based on the following procedures.

### Standard solution

1000 mg/l Folic acid stock standard solution was prepared by dissolving of 1.01 mg folic acid from 98% in 1mL 0.1 N NaOH. Aliquots of this solution was taken to prepare 100-mg/kg intermediate solution from 1000 mg/kg folic acid in phosphate pH 7.0 buffers. Finally, 5 calibration points;0,0.5,1,2,3, and 4, mg/kg was prepared used to calibrate an HPLC UV detector.

### Sample extraction

0.5 g flour sample was extracted with 5 ml of phosphate buffer pH 7.0 (0.25 mol L^− 1^ dibasic sodium phosphate and 0.37 mol L^− 1^ monobasic potassium phosphate). The mixture was shaken for 30 min in a rotational shaker, and centrifuged at 3000 rpm for 15 min. The supernatant was filtered through a 0.22 μm nylon membrane and 20 µl extract was injected.

### Chromatograph conditions

Determination of folic acid was carried out using Shimadzu high performance-liquid chromatography (HPLC), equipped with: solvents degasser, quaternary pump, UV–VIS detector, computer software, column thermostat, and column C-18, (250 × 4.6 mm, 5 μm). The column temperature was maintained at 25 °C. Acetonitrile and acetic acid 1% (20/80 v/v, pH 2.8), isocratic system, was used to separate folic acid, the flow rate was 0.5 ml/min and Injection Volume: 20 µl, Folic acid was detected by a UV detector at a wavelength of 280 nm.

#### Moisture content analysis of flour

The AOAC official method 925.10 was used for moisture content determination AOAC, (2016). Approximately 2 g of the well-mixed test portion was weighed in a cooled and weighted aluminum dish (provided with a cover) that was previously heated to 130 °C. Then, the sample was dried for 2 h in a previously heated oven maintained at 130 ± 3 °C. After that, the dish was covered, transferred to desiccators, and weighted soon after reaching room temperature. The flour residue as total solids and the loss in weight as moisture were reported.

#### Ash content analysis of flour

The AOAC official method 923.05,2016 was used for ash content determination (AOAC, 2016). The ashing crucible was washed with water and HCl and placed in the oven at 1000 °C for 30 min to dry. After 30 min, the hot crucible was placed in the desiccators to cool it to room temperature. Then its mass was weighed using a digital balance, and the mass was recorded as W1. About 3 g of sample was measured and transferred into the crucible, then the mass of the sample and the crucible together was weighed and recorded as W2. The crucible with the sample was placed on the hot plate at a low temperature, to char the sample until it turned black. Then the charred sample was transferred to the furnace at 550 °C for 2 h, and then the crucible was cooled. Then, after cooling in the desiccators for 30 min, the ash and the crucible were measured and recorded as W3 (ash and crucible). The percentage of ash was calculated using the following formula:

$$\mathrm{Ash}\;(\%)\;=\;\frac{\mathrm W3-\mathrm W1}{\mathrm W2-\mathrm W1}\times100$$

#### Fat content analysis of flour

The AOAC official method 2003.05 was used for fat content determination (AOAC, 2016). The aluminum cup with boiling chips was dried in a drying oven at 102 °C for 30 min, and 3 g of well-mixed samples were measured and transferred into thimbles using the thimble support balance. Then a defatted cotton plug was placed on top of the sample and attached with the magnetic ring to hang the thimble on the condenser. Then the condenser was moved to the load position by lifting the right handle to the upper position and the left handle to the lower position, and the thimbles were inserted manually one by one or using the thimble holder.

Both handles were moved to the upper position, and the extraction cup with boiling chips was inserted using the cup holder. The right hand was moved to the middle position to mate the cup with the condenser, and then the left handle (sample load) was moved to the middle position to open the valves for solvent loading. About 70 ml of solvent was added to each cup using the connecter on the top of the extraction unit. The solvent addition kit was connected to a dispenser, and the right handler was lowered to clamp the condenser and cup to the hot plate. The program was started on the control unit by pressing the key, and the sample loading handler was moved to the lowest position. After finishing all the processes, the extraction cups were removed from the extractor, and the extraction cups were dried in a 105 °C oven for 30 min to remove moisture. After drying, the extraction cup was removed from the oven and cooled in a desiccator for 30 min. The extraction cups were weighed immediately after they were taken out of the desiccator.

$$\mathrm{Fat}\;\%\;=\;\frac{\mathrm{Wf}}{\mathrm{SW}}\times100$$

Where: Wf = Weight of aluminum cup after extraction – weight of aluminum cup before extraction and SW = weight of samples.

### Evaluation of the oxidative status of flour

#### Determination of peroxide

Peroxide level was determined after fat extraction from flour using AOAC method number 965.33. (AOAC, 2016). After extraction, the extracted fat was put into a 250 ml glass-stopper Erlenmeyer flask. Then it was swirled in 30 ml of CH_3_COOH-CHCl_3_ (3 volume acetic acid to 2 volume chloroform), and a 0.5 ml saturated KI solution was added. After that, it was left to stand with occasional shaking for 1 min, followed by the addition of H_2_O (30 ml). Finally, it was slowly titrated with standardized 0.01 M Na_2_S_2_O_3_ with vigorous shaking until the yellow is almost gone. Finally, slowly titration was continued by adding 0.5 ml 1% starch solution followed by vigorous shaking, until blue color just disappears. The peroxide value (P0V), was expressed in milli equivalents of active oxygen per kilogram of sample, and it is given by the formula:

$$\mathrm{PV}=\frac{\mathrm V\times\mathrm M\times1000}{\mathrm m}$$

V = the number of ml of the standardized sodium thiosulphate solution used for the test, corrected to take into account the blank test.

M = the exact molarity of the sodium thiosulphate solution used, in mol/l.

m = the weight in g, of the test portion.

#### Standardization

K_2_Cr_2_O_7_ (0.20 g) (previously dried for 2 h at 100 °C) was precisely weighed and placed in a glass-stoppered iodine flask. KI (2 g) was dissolved in 80 ml of chlorine-free water, then swirled in 20 ml of 1 M HCl and immediately placed in the dark for 10 min. Na_2_S_2_O_3_ solution was used for titration, and starch was added to the solution once the majority of the I_2_ had been consumed.

$$\scriptsize \scriptsize \scriptsize \scriptsize \scriptsize \scriptsize \scriptsize \scriptsize \scriptsize \scriptsize \scriptsize \scriptsize \scriptsize \scriptsize \large \scriptsize \mathrm{Molarity}\;(\mathrm{mol}/\mathrm L)\;=\;\frac{\mathrm g\:\mathrm {K}2\mathrm{Cr}207\times1000}{\mathrm{mL}\:\mathrm{Na}2\mathrm S203\times49.032}$$

#### Determination PH of flour

The PH of flour was determined using AOAC Standard Methods of Analysis 20th Edition.

Method 943.02. 10.0-gram flour was weighted into a clean, dry Erlenmeyer flask, and 100 ml of boiled water at 25 degrees Celsius was added and shaken until particles were evenly suspended and the mixture was free of lumps. The mixture was then digested for 30 min by mixing it frequently. The supernatant was decanted into a 250-ml beaker and immediately determined the pH using an electrode and a potentiometer standardized by pH 4.0 and 7.0 buffer solutions.

### Statistical analysis

The results were expressed as mean ± SD, and data was examined using an analysis of variance (ANOVA). The sample analysis was done triplicate.

Tukey’s post hoc multiple comparison was utilized for the observed mean micronutrients in stability during storage, with a significance level of 0.05 (*p* < 0.05). SPSS 26 software were used to analyze the data.

## Results and discussion

### Iron, zinc and folic acid concentration before and after fortification

Table 2 shows the mean iron, zinc, and folic acid content of wheat flour before and after fortification. Before fortification, the wheat flour contained 26.4 mg/kg of iron. But, after the fortification, the amount of iron increased to 56.96 mg/kg.

**Table 2:** The contents of Iron, Zinc, and folic acid in wheat flour before and after fortification

Nutrient	Amount of fortification	Type of wheat flour	
		Unfortified	Fortified
Zinc(mg/kg)	80	13.0 ± 1.7	84.8 ± 7.2
Iron (mg/kg)	30	26.4 ± 1.15	56.96 ± 3.3
FA (mg/kg)	2	0.288 ± 0.16	1.947 ± 0.12

Data are expressed as mean ± standard deviation (SD) *n* = 6

Similarly, the zinc concentration was 13 mg/kg before fortification, while the concentration increased to 84.8 mg/kg after the addition of 80 mg/kg zinc from zinc oxide (99%). The folic acid concentration also increased from 0.288 mg/kg to 1.947 mg/kg after the fortification.

### Iron, zinc and folic acid retention during storage of the fortified wheat flour

The iron and zinc contents were not significantly changed in the fortified wheat flour during the six-month storage period when compared to the zero time as shown in Table 3). The retention of iron and zinc was (96 to 100%).

**Table 3:** Retention of iron, zinc and folic acid in fortified wheat flour

Nutrients	Temperature	Storage time in months			% Retention at 6 months
		0	3	6	
Zinc(mg/kg)	25	84.8 ± 7.19^aA^	81.07 ± 8.5^aA^	81.45 ± 2.5^aA^	96.1^a^
	35	84.8 ± 7.19^aA^	84.04 ± 2^aA^	84.27 ± 0.97^aA^	99.4^a^
	45	84.8 ± 7.19^aA^	84.14 ± 1.05^aA^	84.57 ± 2.7^aA^	99.7^a^
Iron(mg/kg)	35	56.96 ± 3^aA^	54.67 ± 1.9^aA^	54.9 ± 3.3^aA^	96.4^a^
	35	56.96 ± 3.3^aA^	57.8644 ± 2.3^aA^	57.15 ± 2.6^aA^	100.3^a^
	45	56.96 ± 3.3^aA^	55.9233 ± 4.0^aA^	57.27 ± 1.9^aA^	100.5^a^
FA (mg/kg)	25	1.947.3 ± 101.8^aA^	1.878. ±108^aB^	1.709.3 ± 36.5 ^ac^	87.78^c^
	35	1.947.3 ± 101.8^aA^	1.784.3 ± 6.02^bB^	1.506.7 ± 98.1^bC^	77.35^b^
	45	1.947.3 ± 101.8^aA^	1.512.0 ± 22^cB^	1.333.0 ± 57.3^cC^	68.46^a^

Different small letters down column corresponded to significant differences (*p* ≤ 0.05), of the mean between storage temperature in each micronutrient, different capital letter across row correspond to significant differences (*p* ≤ 0.05) of the mean between storage periods., Data are expressed as mean ± standard deviation (SD) *n* = 3

*FA Folic Acid

We found that the iron and zinc contents did not vary over the storage of the six-month period, regardless of the storage conditions. Thus it may be Zinc was added as zinc oxide, which is insoluble and non-reactive, making it highly stable in the dry, low-moisture environment of flour [27]. Iron was added as Sodium Iron EDTA, a chelated compound where iron (Fe³⁺) is bound to ethylenediaminetetraacetic acid (EDTA). This chelation prevents the iron from reacting with other compounds, thereby maintaining its stability throughout the storage period [35].

Similar results reported, in the study conducted in fortified rice which stored at different conidiation of temperatures and humidity [36] and reported as Overtime and regardless of temperature and humidity conditions, iron and zinc concentrations were, in general, similar to baseline, with no significant differences observed.

Study conducted by Alam et al. (2007) favors this study which reported that no significant differences were found in the total iron levels in whole wheat flour samples that had been fortified with premix containing ferrous sulfate, ethylenediamine tetra acetic acid, and folic acid (20.0:20.0:1.5 mg/kg) and stored at room temperature for 60 days. Similar results were reported in fortified flour that contained 30 mg/kg ferrous sulfate, 1.3 mg/kg folic acid, and 40 mg/kg zinc oxide.

This study also supported by another study by Ayelign [37], also reported that storage time at 45 days had no significant effect (*p* >0.05) on the stability of iron in wheat flour fortified with 40 mg/kg of iron sulfate (FeSO4.7H2O). However, another study by Akhtar et al. (2010) reported that the type of iron source significantly influenced iron content during storage and that the degree of fortifying agents had no effect on the concentration of iron during storage.

**Fig. 1:**
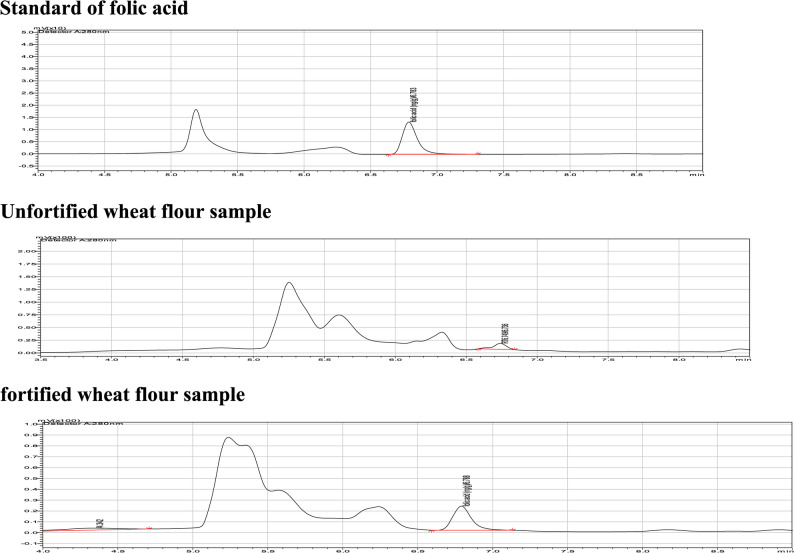
Chromatograms of the blank sample, standard, fortified wheat flour sample

On the other hand, the folic acid concentration was significantly varied by both storage temperature and duration, as shown in Table 3. The percent of retention decreased as the storage temperature increased. After six months of storage at 25 °C, 87.78% of the folic acid was retained. After three- and six-months storage of flour at 35 °C, folic acid retention was 91.63% and 77.37% of the initial amount, respectively. Folic acid retention was also 77.65% and 68.45% of the initial amount after three- and six-months period storage of flour at 45 °C, respectively. In Fig. 1 the chromatograms of the standard, fortified wheat flours are illustrated in the chromatograms.

Similar observation have seen by khamila and reported A 12.8% reduction in folic acid was observed at 25 °C/RH 60% storage after 6 months in fortified maize and The and (24.1%) of folic was reduced in 6- month for the samples stored at 35 °C/RH 75% [38].The change in folic acid might be caused by the prolonged storage duration and the elevated temperature plays a significant part in its oxidative breakdown. This is because Folic is an unstable chemical that is heat sensitive and could possibly undergo oxidative destruction, which can be accelerated by pH, and acidic environments [39]. A reduction in folic acid retention in fortified wheat flour was also reported by [40] with increased temperature and storage time. Another study carried out by youna M et al. (2020), showed higher loss than our reports. Acid (Vitamin B9) flour was stored in permeable paper bags at high relative humidity (85% RH), losses reached 22–53% for folic acid over 6 months [41].

Folic acid losses occurred mainly during the first three months of storage, with 17–19% of the initial folic acid lost after 3 months when flour samples were stored at 65% RH in paper bags, irrespective of storage temperature. When flour samples were stored at 85% RH in paper bags, the folic acid loss after 3 months ranged from 21 to 22% (when stored at 25 °C) to 40–49% when stored at 40 °C [41].

### The moisture content in flour samples during storage

The moisture content of the fortified wheat flour ranged from 11.6% to 12.3%. But it didn’t vary with storage period and storage temperature as shown in Table 4. This finding was supported by [30] in which, moisture content of flour had no insignificance that was stored in a polyethylene bag that did not allow moisture to pass through it.

**Table 4:** Moisture content (%) of fortified wheat flour stored under different temperature

Moisture	Temperature	Storge Time period in months		
		0	3	6
	25	12.3 ± 0.15^aA^	11.87 ± 0.68^bA^	11.83 ± 0.46^cA^
	35	12.3 ± 0.15^aB^	12.3 ± 0.49^bB^	11.8 ± 0.42^cB^
	45	12.3 ± 0.15^aC^	11.69 ± 0.86^bC^	11.61 ± 0.77^cC^

Data are expressed as mean± standard deviation (SD) *n*=3

*Different small letters down column corresponded to significant differences (*p* ≤ 0.05), of the mean moisture between temperature, different capital letter across row correspond to significant differences (*p* ≤ 0.05) of the mean between storage periods

The finding of this study was consistent with a previous study by [30], who reported that the moisture content of wheat samples packed in PET/aluminum bags did not change significantly over time. However, another study by Akhtar et al. (2005) reported the variation in moisture content in fortified flour stored under different storage conditions.

### Evaluation of the oxidative status of the wheat flour

#### Peroxide value (POV) of the wheat flour

Following prolonged storage in a polyethylene bag for six months at temperatures of 25, 35, and 45 °C, samples of fortified wheat flour were examined for changes in the oxidative status of the flour peroxide value, and the results are shown in Table 5.Peroxide value is an essential criterion to determine the primary oxidation of fats The Peroxide value is a marker of the initial phases of lipid oxidation, indicating an accumulation of hydroperoxides [42, 43].The peroxide value in the wheat flour significantly changed during the storge of the six-month period when compared to the zero months. The peroxide value in the baseline was not detectable and was regarded as zero. The peroxide value at the 3-month, highest concentration (0.10916 ± 9.1 milli eq/kg of fat) was recorded at 45 °C, and the lowest concentration (0.09346 milli eq/kg of fat) was recorded at 25 °C. At the end of 6 months, the peroxide content was 0.12203 ± 5.8, 0.12834 ± 10.0, and 0.12834 ± 10.0 milli Eq/kg of fat at 25, 35, and 45 °C, respectively.

The peroxide value in the fortified wheat flour increased throughout the storage period. The peroxide levels of flour samples that were stored at 45 °C were higher, followed by 35 °C and 25 °C.

The concentration of peroxide in wheat flour was significantly affected by both temperature and storage time. This implies that higher temperature and long storge accelerated lipid oxidation. Similar result was reported in the study of Hong-Jam which is stored at 5,25,35 and 45 °C [42]. Peroxide value (POV) significantly increased at higher temperatures which implies accelerated lipid oxidation at higher temperature(45 °C).

Similar result was also observed, in another study that carried out by (Lingyu Qu et al. (2024)

According to the study [44],high temperatures have been shown to hasten the development of peroxide and oxidative rancidity and lower the POV of Japonica Brown Rice Was recorded, at lower temperatures (15 °C), which were stored at 15 °C, 20 °C, and 25 °C.

**Table 5:** Peroxides value (milli eq/kg of fat) of the wheat flour during entire storage period

Peroxide (*milli* eq/kg of fat)	Temperature	Storage time in months		
		0	3	6
	25	0.00^aA^	0.09346 ± 5.0^aB^	0.12203 ± 5.8^Ac^
	35	0.00^aA^	0.09660 ± 5.3^aB^	0.12834 ± 10.0^aC^
	45	0.00^aA^	0.10916 ± 9.1^bB^	0.14844 ± 9.9^bC^

*Different small letters down column corresponded to significant differences (*p* ≤ 0.05), of the mean in each peroxide between temperature, different capital letter across row correspond to significant differences ((*p* ≤ 0.05) of the mean between storage periods. Data are expressed as mean± standard deviation (SD) *n *=3

Peroxides value in this study was comparable with study done by Rebellato and others and reported fortified flour by iron sodium EDTA contain 9.6–12.5 micro equivalent of hydrogen peroxide/kg of sample which is stored for 4 month periods [35].

However, Peroxides value in this study was lower than reported by Huma et al. (2007), which showed that the POV considerably increased from 0.8 to 1.6 milli Eq/kg following storage of flour for 42 days. The variation in sodium EDTA’s fortification of iron in its ferric state could be the reason.

In this study, the peroxide value in fortified wheat flour was below the Codex Alimentarius limit of 10 milli Eq per kilogram of fat, which is the standard value used to reject products. This study showed that all storage temperatures for fortified flour in polyethene bags had acceptable peroxide values.

#### PH value of the wheat flour

Following six months of storage in a polyethylene bag at temperatures of 25, 35, and 45 °C, the flour’s acidity value was determined in terms of pH, and the results are shown in Table 6.

**Table 6:** The PH contents of the wheat flour during storage

PH	Temperature	Storage time in months		
		0	3	6
	25	6.23 ± 0.12^aA^	6.00 ± 0.06^aB^	5.66 ± 0.23^aC^
	35	6.23 ± 0.12^aA^	5.89 ± 0.1^bB^	5.65 ± 0.06^aC^
	45	6.23 ± 0.12^aA^	5.79 ± 0.06^cB^	5.38 ± 0.37^bC^

Data are expressed as mean± standard deviation (SD) *n*=3

*Different small letters down column corresponded to significant differences *p* ≤ 0.05), of the mean PH between temperature, different capital letter across row correspond to significant differences ((*p* ≤ 0.05) of the mean between storage

PH measure of how acidic or basic a solution is and indirectly related to acid value. Acid value reflects the concentration of free fatty acids, thereby indicating the degree lipid hydrolysis [42]. In this finding value ranges from 5.38 to 6.23, as shown in Table 6.

The PH value at the 3-month, highest (6.00 ± 0.06) was recorded at 25 °C, and the lowest (0.79 ± 0.06) was recorded at 25 °C. At the end of 6 months, the PH content was 5.66 ± 0.23, 5.65 ± 0.06, and 05.38 ± 0.37 at 25, 35, and 45 °C, respectively.

The PH value in the fortified wheat flour decreased throughout the storage period. The peroxide levels of flour samples that were stored at 45 °C were lowest, followed by 35 °C and 25 °C.

The PH value in the wheat flour decreased significantly in both temperature and time period during a six-month storage period, with rates increasing as temperature and retention time increased.

The creation of free fatty acids and the hydrolysis of lipids during storage result in an increase in acidity value or a fall in PH. Our findings are the same with those of a prior study by Miranda and El-Dash (2002), who stated that they monitored the stability of germinated whole wheat flour during 6 months of storage and discovered that the acidity values increased during the evaluation period, for both control and germinated samples, and that this occurrence was attributed to the gradual hydrolysis of triacylglycerol with the formation of free fatty acids.

Similar finding was reported in the study of Hong-Jam which is stored at 5,25,35 and 45 °C [42],acid value (AV) significantly increased at higher temperatures which implies accelerated lipid oxidation at higher temperature(45 °C).

When different iron compounds, primarily NaFeEDTA, were used in the process of fortification, the acidity of the wheat flour during storage was marginally impacted, which had a detrimental impact on the flour’s quality [35]. But according to the Ethiopian Standard Agency’s suggestion of ES 6132:2021: fortified wheat flour specification [28] in our investigation, the PH value in the fortified wheat flour complied with the specification. This outcome demonstrated that enriched flour stored in polyethene bags at all storage temperatures had acceptable PH values.

### Quality control and data validation

All calibration curves have R^2^ values greater than 0.999 for sample analysis, with all calibration errors less than 5%. The peak for folic acid appeared in an isolated form with a retention period of 6.8 min. The folic acid concentration was determined using external standardization (standard solutions of 0.0–4 mg/kg), with the analytical curve exhibiting satisfactory linearity in the predetermined concentration bands. By adding the standards to wheat flour, recovery tests for folic acid were carried out. Recovery of folic acid was showed in Table 7.

$$Recovery\;=\;\frac{final\;wheat\;flour\;of\;vitamin\;obtained\;-\;wheat\;of\;vitamin\;un\;spiked}{spiked\;con\;of\;vitamin.}\ast\;100$$

The recovery value of folic acid at two points or at 1 mg/kg and 2 mg/kg, was greater than 91.19 and 93.9%, respectively. These findings showed that the validity and reliability of the data.

**Table 7:** The value of recovery value of folic acid in wheat flour

Spiked level folic acid in flour(ug/100 g)	Final con of vitamin obtained(ug/100 g)	Un spiked con Folic acid(mg/kg)	% Of Recovery
200	216.5	0.288	93.9
100	120.0	0.288	91.19

Result expressed mean and *n* = 4

To ensure quality control, certified reference material (CRM) for wheat flour with the code 1567b, obtained from National Institute of Standards and Technology in the U.S. A department of commerce, was used for the analysis quantification of zinc and iron. The certified values of the CRM were iron: 14.11 ± 0.33 mg/kg and zinc: 11.61 ± 0.26 mg/kg. the value of iron and zinc, our results for the CRM, presented in Table 8, shows no significant difference((*P* = 0.05) from these certified values, confirming the accuracy of our methods. This CRM was analyzed alongside with every batch of samples throughout the study.

**Table 8:** The value of iron and zinc in verified reference material of wheat

Micro content of C.*R*.M of flour (mg/kg)		True value of C.*R*.M flour (mg/kg)	
Zinc	Iron	Zinc	Iron
12.59 10.88 11.70 12.89	14.03 14.53 15.60 14.24	11.61 ± 0.26	14.11 ± 0.33
Mean = 12.01 ± 1.08	Mean = 14.8 ± 0.81		

Result expressed mean ± SD. *n* = 4

## Conclusion and recommendation

The findings demonstrate that zinc and iron showed no significant losses at any stored temperature; however, folic acid levels decreased significantly with increasing temperature and time period. Peroxide value and the pH value significantly changed with increasing temperature and time period, but values were within the Ethiopian quality standards.

Based on the finding result in this research the following are recommended, the storage on physical and sensory characteristics (colour, texture, taste and smell), analysis of microbiological loads during storage, and the retention phytates which have a direct association with the bioavailability of iron and zinc has to be studied in the future. Additional research will be required to evaluate and clarify the separate and combined effects of iron, zinc, time, and temperature on folic acid stability, we highly advise that future studies include control groups with single fortification (iron-only, zinc-only, and folic acid-only).

### Limitation Study

The study’s scope is limited even if it provides valuable data by looking at a single packing type (polyethylene bags) in a realistic setting at various temperatures. In order to properly evaluate the retention of micronutrients (iron, zinc, and folic acid) in fortified white wheat Ethiopian flour, future studies should include controlled humidity variables and alternate packing materials.

## Supplementary Information

Supplementary Material 1.

## Data Availability

The datasets analyzed for this study are available with the corresponding author, which can be accessed on reasonable request.

## Abbreviations

AOAC: Association of American Chemistry Society
EPHI: Ethiopian public health institute
FA: Folic acid
FDRE: Federal demographic republic of Ethiopia
FMOH: Federal ministry of health
IZNCG: International Zinc Nutrition Consultative Group, MNM: Micro nutrient malnutrition
MPAES: Micro plasma atomic emission spectroscopy
NaFeEDTA: Sodium iron (iii) ethylenediaminetetraacetate
NTDs: Neural Tube defects
PH: Power of hydrogen
MG/KG: Part per million
ZnO: Zinc oxide
