# Supplementary material for: Evaluation of the iron, zinc, and folic acid stability in fortified wheat flour storage and its impact on quality indicators
Source: BMC Nutr. 2025 Dec 18;11:226. doi: 10.1186/s40795-025-01213-z (PMC12751853; doi:10.1186/s40795-025-01213-z)
Supplement: Supplementary file 1 — Supplementary Material 1. [file 40795_2025_1213_MOESM1_ESM.docx]

**Calibration curve of iron analysis from flour**

**Calibration curve of zinc analysis from food**

**Calibration curve of folic acid analysis from food.**
